# Plagiarism

**Published:** 1882-12

**Authors:** Ad. Lippe

**Affiliations:** Philadelphia


### PLAGIARISM.

The President of the American Institute of Homœopathy stands accused of having been guilty of plagiarism when he delivered his address before the Institute. Said address is in print and was freely distributed in Louisville, Ky. A Professor Mathews, M. D., had also delivered an address on the 10th of February, 1882, and he accuses the President of the American Institute of plagiarism, in the *Courier-Journal*, Louisville, July 21st, 1882, and proves his charge by publishing some extracts of both his and the American Institute's President's addresses. On July 22d, 1882, there appears in the same journal "A Card from Dr. Breyfogle," obviously for the purpose of setting aside the grave charge made by Professor Mathews. Had Dr. Breyfogle committed the act with which he is charged, merely as a member of the profession we should certainly not have taken any notice of the affair, but if a president of a representative body is so accused, and if the members of such a body, after such charges are made, remain silent, the disgrace of such a charge, if it is found correct, falls upon the whole associated body. After carefully comparing the original address of Professor Mathews, as published in the Louisville *Courier-Journal* of February 10th, and also Dr. Breyfogle's address, as published by the Institute, we now give first Professor Mathews' charge and then

Dr. Breyfogle's card in answer to it, finding them to fully correspond with the original:

The following are Professor Mathews' remarks:

"Great operations in surgery which were regarded with awe by the physicians and dread by the patient are to-day performed with comparative ease and remarkable success. It has been but a few months ago that the medical world was amazed at the report of several cases of resection of the stomach, and yet Billroth and his assistant have performed the operation some half-dozen times and clearly demonstrated the advisability of the same under certain conditions. Just prior to this report Czerny gave a detailed statement of these cases in resection of the intestines; in one subject six and one-half feet were removed. Since his report a Kentucky surgeon has gone him one better (or one-half better), and successfully removed *seven* feet of intestines. You are aware of the fact that both the spleen and the womb have been removed with success. A few days ago Dr. A. C. Post, of New York, took out all the parotid gland, and Mr. Walter Whitehead, of Manchester, England, lately performed the triple operation of gastrotomy, tracheotomy, and excision of the tongue with perfect results. Dr. T. G. Thomas and others have extirpated the kidney and a trans-Atlantic brother has taken out the trachea.

"Enucleation of the ovaries is of frequent occurrence and the aspiration of the different organs almost a daily thing.

"You know with what frequency the operations of ovariotomy, lithotomy, cölotomy, the tying of large arteries, the amputation of limbs, transfusion of blood, trephining, extirpation of cancer, etc., are performed, and to these may be added laparotomy and gastrotomy. Very often is it that we hear of physicians, in a secluded country town it may be, performing one or many of the operations of major surgery. The reason of it is that surgery is to-day studied from a scientific standpoint and its rules

The following is a portion of Dr. Breyfogle's address, delivered four months later:

"Great operations in surgery which were regarded as impracticable are to-day performed with comparative ease and remarkable success. It is but a few months ago that the medical world was amazed at the report of several cases of resection of the stomach, and yet Billroth and his assistants have performed the operation some half dozen times and clearly demonstrated the advisability of the same under certain conditions. Just prior to this report Czerny gave a detailed statement of these cases in resection of the intestines; in one subject six and one-half feet were removed. The credit of having first performed this difficult and dangerous operation is due and should be given to the late Dr. Beebe, of Chicago, a homœopathic physician of great learning and a surgeon of rare ability, for a full report of which case I refer you to the *New York Transactions*, 1869, page 169.

"You are aware of the fact that both the spleen and uterus have been successfully removed. A few months ago Dr. A. C. Post, of New York, enucleated the parotid gland, and Mr. Walter Whitehead, of Manchester, England, lately performed the triple operation of gastrotomy, tracheotomy, and excision of the tongue with perfect results. Dr. J. H. McClelland, of Pittsburg, and others have extirpated the kidney and a trans-Atlantic surgeon has taken out the trachea. Enucleation of the ovaries is of frequent occurrence and the aspiration of the different organs almost a daily practice. To the frequent operation of ovariotomy, lithotomy, colotomy, the tying of large arteries, the amputation of limbs, transfusion of blood, trephining, extirpation of cancer, etc., may be added those of laparotomy and gastrotomy.

"In other special departments the advancement has been equally great. The operation for cataract, once so are definite and fixed. In the special departments the advance has been marked. I am told by a colleague that the operation for cataract, which at one time was regarded with such disfavor, is now one of the most successful known to surgery. It was my pleasure lately to witness the complete restoration of the voice of a young lady friend in whom it had been lost for several months. The operations upon and the treatment for diseases of the ear are successfully practiced. Many of us have seen those who could not stand upon their feet be made to walk by the skill of the surgeon. Thus, verily, do the blind see, the deaf hear, the dumb speak, and the lame walk by our aid."

difficult, is now one of the most successful known in surgery, and in diseases of the ear and throat, equal proficiency has been made. Verily do the blind see, the deaf hear, the dumb speak, and the lame walk through the aid of the skillful surgeon."

A CARD FROM DR. BREYFOGLE.

*To the Editor of the Courier-Journal:*

Statistical facts and reports are common property, and Dr. Mathews or no other physician can claim any originality in reciting them. In that part of my address where any mention is made of surgery I have endeavored to correct an impression made in Dr. Mathews' address in regard to "Czerny" being the first to remove six and one-half feet of the intestines, giving credit where it belongs, viz.: to the late Dr. Beebe, of Chicago, a homœopathic surgeon, hence the similar language. Those who have read the address will know that no *originality* in statistical reports is pretended, nor has Dr. Mathews any more claim to it than myself, he having obtained his information from the *Résumé of Medical Science*, published in 1882. The fact that the address was publicly delivered, and that copies were sent to each allopathic physician in the city (Dr. Mathews included), does not look as if "plagiarism" had been attempted. How would it have sounded to have said: "Dr. Mathews has said 'that Dr. A. C. Post has enucleated the parotid gland'"? I am quite sure a little reflection will convince any fair-minded person that "statistics" are common property. The publicity given to a few lines would naturally excite interest in the address proper, and those who read it can easily see the connection in which those "statistics" were mentioned. If any thoughts or ideas are discovered as having been "plagiarized," then will it be proper to make such charges as were made public this morning. To the curious, I will state that a copy of the address will be mailed to any one making application. Respectfully,

WM. L. BREYFOGLE.

*Comments.*—It would give us much pleasure if we could find some defense which could possibly clear the President of the American Institute from this grave charge. What is "plagiarism," of which Dr. Breyfogle, as President of the American Institute of Homœopathy, stands accused before the world? Swift defines "Plagiarism" as "the act of purloining another man's literary works, or introducing passages from another man's writings and putting them off as one's own; literary theft."

It is here in evidence that passages from another man's writings were introduced by Dr. Breyfogle in his address. In his defense he is still more unfortunate; he claims to have only copied statistical reports; but just what Professor Mathews intended to become statistical reports Dr. Breyfogle professes to correct by giving the late Dr. Beebe the credit claimed for Dr. Czerny; and still more unfortunate is it that Dr. Breyfogle refers to the professedly statistical reports as having been taken from the *Résumé of Medical Science*, published in 1882—very unfortunate indeed. If such a work did exist as the one referred to—the *Résumé of Medical Science* published in 1882—these statistical reports could hardly have been utilized by Professor Mathews on the eleventh day of February, 1882. But it is still more unfortunate that search has been made diligently for this *Résumé of Medical Science*, published in 1882, and no such a work can be found!

The evidence is before us, and if we, as members of the Institute, were asked to enter our verdict, we would unhesitatingly find Dr. Breyfogle—Guilty of Plagiarism.

*Philadelphia, September 4th*, 1882. AD. LIPPE, M. D.

